# Influence of Vitamin D on Developmental Defects of Enamel (DDE) in Children and Adolescents: A Systematic Review

**DOI:** 10.3390/nu17081317

**Published:** 2025-04-10

**Authors:** Paula Piekoszewska-Ziętek, Karolina Spodzieja, Dorota Olczak-Kowalczyk

**Affiliations:** Department of Paediatric Dentistry, Medical University of Warsaw, 02-091 Warszawa, Poland; ppiekoszewska@wum.edu.pl (P.P.-Z.); dorota.olczak-kowalczyk@wum.edu.pl (D.O.-K.)

**Keywords:** vitamin D, vitamin D deficiency, MIH, developmental enamel defects, HSPM

## Abstract

**Background/Objectives:** This systematic review aims to investigate the potential association between vitamin D levels and the occurrence of developmental enamel defects (DDE) in children, including conditions like molar–incisor hypomineralization (MIH) and hypomineralized second primary molars (HSPMs). DDEs, which occur during tooth development, can result in significant aesthetic and functional issues, and their exact etiology remains unclear, with both genetic and environmental factors contributing. Among environmental factors, vitamin D deficiency has been proposed as a possible risk factor, given its role in enamel mineralization. **Methods:** A thorough literature search was conducted in PubMed, Scopus, and Embase. The search strategy included terms such as “vitamin D”, “vitamin D deficiency”, “developmental defects of enamel”, “enamel hypoplasia”, “molar-incisor hypomineralization”, and “hypomineralized second primary molars”. Studies were included if they were original human observational research (cohort, case–control, or cross-sectional) conducted in children under 18 years of age or involving maternal–child cohorts. Ten studies were included in the analysis, with a total of 15,891 participants. The primary data extracted from the selected studies included the following: study design, participants’ age, sample size, vitamin D status in relation to developmental defects of enamel, and statistical significance **Results:** The findings were mixed, with only a few studies suggesting a significant association between low vitamin D levels and the presence of DDEs. Specifically, one study found a link between insufficient maternal vitamin D levels during pregnancy and an increased number of teeth affected by MIH in children. However, the majority of the studies did not report a significant association. **Conclusions:** This review concludes that while there is some evidence to suggest a possible relationship between vitamin D and DDEs, more research is needed to confirm these findings and better understand the underlying mechanisms.

## 1. Introduction

Developmental enamel defects represent a significant issue in the field of pediatric dentistry. They are clinically important due to their impact on aesthetic appearance, dental sensitivity, dentofacial abnormalities, and a higher risk of developing dental caries.

Unlike bone, enamel and dentin lack the ability to remodel. Disruptions in ameloblast function during tooth development lead to permanent defects. Developmental enamel defects (DDEs) are classified into three types: demarcated opacity, diffuse opacity, and hypoplasia. Opacities, which alter enamel translucency, are commonly referred to as hypomineralization defects. Hypoplasia results from an imbalance during the formation of the enamel matrix, whereas hypomineralization arises from an imbalance during enamel mineralization [1,2,3].

Dental enamel defects arise from a complex interplay of genetic and environmental factors. While genetics lay the foundation for enamel formation, environmental influences during critical developmental windows can significantly affect enamel quality and integrity. Over 115 genetic conditions have been identified that affect amelogenesis, the process of enamel formation, leading to variations in enamel quantity and mineralization. These genetic factors can result in hypoplastic (reduced enamel amount) or hypomineralized (defective enamel quality) enamel phenotypes, highlighting the crucial role of genetic predisposition in enamel development.

In addition to genetic factors, environmental influences can disrupt enamel development at various stages, leading to developmental defects of enamel (DDEs). These factors include excessive fluoride intake during enamel development, childhood illnesses, prenatal factors, and dietary components.

Excessive fluoride can cause dental fluorosis, a condition characterized by enamel opacities and hypomineralization. Fluoride exposure during critical periods of tooth formation can significantly alter enamel structure, leading to varying degrees of severity depending on the timing and amount of exposure. Childhood illnesses, systemic conditions that affect calcium metabolism or disrupt specific biological pathways, such as the aryl hydrocarbon receptor nuclear translocator (ARNT) pathway, can lead to DDE. Illnesses during the critical phases of tooth development can interfere with mineralization, resulting in defects that compromise the structural integrity of the enamel.

Maternal health issues, medication use during pregnancy, low birth weight, and prematurity are all recognized as adverse factors that can negatively affect enamel development in offspring. Disruptions during prenatal and perinatal periods can have lasting consequences on enamel formation, making these stages particularly vulnerable to environmental influences. Nutritional deficiencies or imbalances during tooth development, especially those affecting key minerals like calcium, vitamin D, and phosphorus, can have a profound impact on enamel formation and quality. A deficiency in these essential nutrients can impair mineralization, leading to weaker and less durable enamel, which is more susceptible to defects [4,5].

Furthermore, adequate nutrition, including sufficient levels of calcium, magnesium, phosphorus, and vitamin D, is essential for maintaining good oral health. Deficiencies in these minerals can lead to impaired absorption, increased bleeding tendency, bone resorption, looseness, and premature tooth loss. Notably, taking calcium without magnesium has been associated with softer dental enamel, which is more susceptible to decay. Additionally, vitamin D exerts anti-inflammatory effects and aids in calcium absorption and bone remodeling, further supporting its role in enamel mineralization [6].

Among DDEs, molar–incisor hypomineralization (MIH) and hypomineralized second primary molars (HSPMs) are common, affecting approximately 13–14% of children, according to recent meta-analyses. They are qualitative developmental defects of enamel that occur during the maturation phase [7,8]. They present clinically—demarcated opacities from whitish to brownish color in the milk secondary molars and permanent first molars, but the permanent incisors are often also involved. As a result, these teeth may experience significant sensitivity, post-eruptive tissue deterioration, and an increased risk of caries. Opacities on anterior teeth are less likely to cause functional issues but can lead to cosmetic and psychosocial concerns [9,10].

Although the etiology of DDEs is unknown, among the potential causes, the following stand out: prenatal factors, childhood illnesses, renal failure, fever episodes, early antibiotic use, or respiratory illnesses requiring corticosteroid therapy [11,12].

A significant advancement in understanding the pathogenesis of MIH is the “mineralisation poisoning” model proposed by Hubbard et al. in 2021 [13]. This model suggests that MIH results from the localized exposure of immature enamel to serum albumin during tooth development. Serum albumin binds to enamel-mineral crystals, inhibiting their growth and leading to the formation of chalky opacities with distinct borders. This mechanism shifts the focus from previously held beliefs that primarily implicated systemic disturbances affecting ameloblasts (enamel-forming cells) to a model emphasizing direct interference with enamel mineralization by serum albumin. The mineralization poisoning model offers new avenues for research into the causes and prevention of MIH [13].

There is ongoing debate about the potential link between vitamin D deficiency and conditions such as molar–incisor hypomineralization (MIH) and hypomineralized second primary molars (HSPMs). Vitamin D plays a vital role in maintaining calcium homeostasis, which is essential for bone and dental tissue development. Beyond its established role in skeletal health, growing evidence supports its involvement in enamel formation, as vitamin D receptors are present in both dental and bone cells, where they mediate mineralization processes [14,15]. Early deficiency in vitamin D has been associated with several adverse oral outcomes, including delayed tooth eruption, enamel defects, increased caries risk, gingival inflammation, tooth loss, and impaired growth [16]. These effects are attributed to the influence of vitamin D metabolites on the function of ameloblasts and odontoblasts [16]. Furthermore, studies suggest that inadequate vitamin D levels can interfere with enamel and dentin mineralization, while excessive levels may also alter tooth calcification. Maintaining adequate vitamin D levels during critical periods of development is therefore important for optimal enamel integrity and may help reduce the risk of dental caries [17,18]. Enamel hypoplasia and other developmental defects of enamel increase the risk of early childhood caries by weakening the enamel and promoting plaque retention. Prospective studies from Canada have shown that low maternal vitamin D levels during pregnancy are associated with a higher risk of enamel defects and dental caries in children. Schroth et al. [19] found a significant link between prenatal vitamin D deficiency and increased caries in infants. Subsequent studies confirmed that prenatal vitamin D supplementation may reduce caries risk [20] and identified low vitamin D as a determinant of enamel hypoplasia [21].

This systematic review provides a comprehensive summary of current evidence on the association between prenatal and postnatal vitamin D levels and the occurrence of enamel defects in children.

## 2. Materials and Methods

### 2.1. Protocol

**Figure 1:**
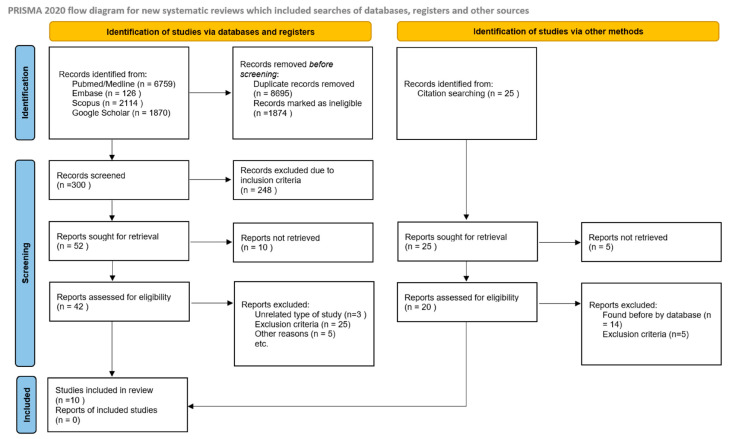
Prisma flow diagram.

This systematic review was conducted in accordance with the Preferred Reporting Items for Systematic Reviews and Meta-Analyses (PRISMA) 2020 guidelines (https://www.prismastatement.org accessed on 16 January 2025). The PRISMA flow diagram is presented in Figure 1. The review protocol was registered in the PROSPERO database (Registration ID: 1016586).

### 2.2. Study Question

Is there an association between vitamin D status and the occurrence of developmental defects of enamel of primary or permanent teeth in children?

### 2.3. PECO Framework [22]

The study population was children with all types of dentition (under 18 years). The exposure was prenatal and/or postnatal vitamin D status in children with developmental defects of enamel (DDEs) of primary and permanent teeth, while the comparator was vitamin D status in children without developmental defects of enamel of primary and permanent teeth. The outcome showed the association of vitamin D status with DDEs.

### 2.4. Inclusion Criteria

- Original human research with an observational methodological study design (prospective cohort, case–control, cross-sectional studies).
- Studies performed on children aged under 18 years, with primary, mixed, or permanent dentition, or longitudinal studies on both pregnant women and their children.
- Articles published in English, with full text availability.

### 2.5. Exclusion Criteria

- Animal studies, in vitro studies, reviews, editorials, commentaries, abstracts, or research protocols.
- Studies performed on children with systemic disease requiring regular medical care or chronic medication intake; children with physical or mental disabilities; or children with developmental abnormalities of the oral and maxillofacial region.
- Studies that include adults above 18 years.
- Articles published in languages other than English.

### 2.6. Search Strategy

The electronic search was conducted on 16 January 2025. PubMed/Medline, Scopus and Embase databases were used to search for applicable studies. Additionally, Google Scholar was screened for any relevant articles. Furthermore, the reference list from retrieved full-text articles was inspected and searched manually to identify additional studies.

We used the following search strategy and its modifications to search for eligible articles:

“vitamin D” OR “vitamin D level” OR “vitamin D deficiency” OR “vitamin D” [MeSH] OR “vitamin D deficiency” [MeSH] AND “developmental defects of enamel” OR “developmental defects of enamel” [MeSH] OR “enamel hypoplasia” OR “dental enamel hypoplasia” OR “dental enamel hypoplasia” [MeSH] or “molar-incisor hypomineralization” OR “molar hypomineralization” [MeSH] OR “Hypomineralized Second Primary Molars”.

Title and abstract screening was conducted independently by two reviewers (PPZ and KS) based on the predefined eligibility criteria. Full texts of potentially eligible studies were then retrieved and assessed independently by the same reviewers. Any discrepancies during the selection process were discussed and resolved in consultation with a third reviewer (DOK). Studies not meeting the inclusion criteria were excluded.

### 2.7. Data Extraction

Data collection was conducted independently by two reviewers (KS and PPZ), and the results were compared to assess data accuracy. Any discrepancies were resolved through discussion between the reviewers. The primary data extracted from the selected studies included study details (author, year, and country of publication); study design; participants’ age; sample size; vitamin D status in relation to developmental defects of enamel; statistical significance; and conclusions.

### 2.8. Risk of Bias Assessment

The included studies were assessed for the potential risk of bias using the Newcastle–Ottawa Scale (NOS), a tool designed to evaluate the quality of non-randomized studies [23]. NOS employs a three-dimensional assessment approach, examining the selection of participants (0–4 stars), comparability of study groups based on controlled relevant factors (0–2 stars), and outcome assessment (0–3 stars). The overall study quality is classified according to the following thresholds: good quality (≥7 stars), fair quality (4–6 stars), and poor quality (0–3 stars).

## 3. Results

Ten original articles were included in this systematic review. The PRISMA Flow Diagram is presented in Figure 1.

### 3.1. Primary Characteristics of Individual Studies

The included studies investigated the association of vitamin D status with the occurrence of developmental defects of enamel in children with primary or permanent dentition. The main characteristics of the included studies in the qualitative synthesis are presented in Table 1. A total of 15,891 subjects (children or pregnant mother–child pairs) were recruited in the included 10 primary studies. The involved studies were published between 2015 and 2024. They were performed in Canada [21], Scandinavia [24,25,26,27], Finland [28], New Zealand [29], Germany [30], USA [31], and The Netherlands [32].

The findings of the included studies based on differences in vitamin D status between participants with or without developmental defects of enamel are summarized in Table 2. Eight out of ten cited papers did not report any significant association between vitamin D status and the development of enamel defects. Overall, the findings from the included studies were mixed. While most studies did not find a statistically significant association between vitamin D status and the presence of developmental defects of enamel (DDE) [21,27,29,31,32], a few indicated potential links—particularly between prenatal vitamin D insufficiency and an increased number of MIH-affected teeth [25]. One study found a significant inverse association between serum 25(OH)D levels and MIH occurrence [30], while others reported trends without statistical significance [24,26,28]. Differences in study design, vitamin D assessment methods, outcome definitions, and populations likely contributed to the variability in results.

### 3.2. Risk of Bias Assessment

For the risk of bias evaluation, The Newcastle–Ottawa Scale (NOS) was used. The included studies were scored from five to eight stars. Three studies were classified as fair quality, and the remaining seven as good quality. The biggest problems were observed with selection in cohort studies, as many of the research examples were continuations of vitamin D-related RCTs conducted within a single center, so the study selection does not fully reflect the population and the adequacy of the follow up of the cohorts was inconsistent. The details of NOS assessment are presented in Table 3.

## 4. Discussion

This systematic review examined the association between vitamin D status during prenatal and early childhood periods and the occurrence of developmental defects of enamel in children. Understanding this relationship is clinically relevant, as DDEs can compromise tooth structure, increase caries risk, and negatively impact oral health outcomes [16,17]. Despite a growing interest in the role of vitamin D in mineralized tissue development, the current body of evidence remains inconclusive. While some studies suggest a potential link, particularly in specific forms such as MIH, the findings are not consistent across study designs and populations. These inconsistencies likely reflect methodological variations, differences in exposure timing and vitamin D assessment, and the multifactorial etiology of enamel defects.

Vitamin D plays a crucial role in the body, influencing the function of many systems, including the regulation of mineral homeostasis. It is actually a steroid hormone, not a vitamin. The biologically inactive precursors, vitamin D2 (ergocalciferol) and vitamin D3 (cholecalciferol), undergo metabolic activation in the liver and kidneys. Vitamin D2 is mainly derived from dietary sources such as oily fish and fortified dairy products, while vitamin D3 is synthesized in the skin upon exposure to ultraviolet B radiation (230–313 nm), which triggers the activation of 7-dehydrocholesterol in skin cells. Both forms are first hydroxylated in the liver, producing 25-hydroxyvitamin D (25(OH)D or calcidiol), a prohormone that is later converted in the kidneys into the biologically active 1,25-dihydroxyvitamin D (1,25(OH)2D or calcitriol). Serum 25(OH)D levels serve as a key marker of vitamin D status in the body. The conversion of 25(OH)D into its active form is regulated by parathyroid hormone (PTH), along with calcium and phosphate concentrations. Vitamin D primarily facilitates calcium absorption in the intestines, maintaining optimal serum calcium levels necessary for bone formation and maintenance. However, vitamin D receptors are widely distributed across various tissues throughout the body [33,34].

Vitamin D plays a crucial role in dental health, particularly in the development and mineralization of tooth enamel. Research indicates that vitamin D obtained from various sources—sunlight exposure, dietary intake, and supplements—can influence enamel development.

Limited exposure to sunlight can reduce the body’s ability to synthesize vitamin D, which may contribute to oral health problems, including enamel defects. Sunlight remains a key factor in maintaining adequate vitamin D levels that support tooth mineralization and general oral health. In addition to sun exposure, vitamin D can be obtained from dietary sources such as beef liver, fish, egg yolks, cheese, and butter. Research suggests that increased maternal vitamin D intake during pregnancy may lower the risk of dental caries in children. For instance, one study conducted among Japanese children reported a protective effect of higher maternal vitamin D intake against caries development. Furthermore, vitamin D supplementation during pregnancy has been shown to help prevent enamel defects in offspring. A randomized clinical trial found that high-dose prenatal vitamin D supplementation reduced the risk of enamel defects in children at age six by approximately 50% [35,36,37]. The Endocrine Society in the USA provided guidelines for treating and preventing vitamin D deficiency. They recommended maintaining serum 25(OH)D levels above 30 ng/mL (>75 nmol/L), with an optimal range of 40–60 ng/mL (100–150 nmol/L) [38]. Additionally, they advised that infants up to 1 year should receive 400–1000 IU/day (10–25 mg), children over 1 year should receive 600–1000 IU/day (15–25 mg), and all adults should aim for 1500–2000 IU/day (37.5–50 mg) [28], while the European guidelines suggest using vitamin D supplements to achieve and maintain an optimal 25(OH)D concentration within the range of 30–50 ng/mL (75–125 nmol/L) [39].

Numerous studies in the literature have suggested that vitamin D may help prevent the onset and progression of dental caries. Mellanby et al. found a link between vitamin D supplementation and a reduced risk of caries [40]. Vitamin D during the early years of life might serve as a preventive measure against dental caries, as demonstrated in a study by Hujoel et al. [41]. A study titled “Untreated caries and serum vitamin D levels in children and youth of the United States: NHANES 2013–2014” analyzed data from 3072 participants aged 1 to 19 years. The results showed that lower serum vitamin D levels were linked to a higher prevalence of untreated dental caries in children aged 1 to 11 years. Specifically, in children aged 1 to 5 years, vitamin D levels between 25 and 49.9 nmol/mL and below 25 nmol/mL were associated with increased odds of untreated caries. In children aged 6 to 11 years, vitamin D levels between 50 and 74.9 nmol/mL were similarly connected to higher odds of untreated caries. However, no significant associations were observed in adolescents aged 12 to 19 years [42].

Another study performed by Schroth et al. (2016) examined the relationship between vitamin D status and dental caries in Canadian school-aged children. The research utilized data from the Canadian Health Measures Survey, focusing on 1017 children aged 6 to 11 years. The findings indicated that 56.4% of the participants had experienced dental caries, with an average caries score of 2.47. Multiple linear regression analysis revealed that lower serum concentrations of 25-hydroxyvitamin D (25(OH)D) were significantly associated with higher caries scores. Specifically, vitamin D levels below 75 nmol/L were linked to increased caries experience. Additionally, factors such as not brushing twice daily, lower household education levels, and fewer annual dental visits were also associated with higher caries scores. The study suggests that improving vitamin D status in children may serve as an additional preventive measure against dental caries [43]. In contrast, Herzog et al. concluded that they did not find a significant correlation between serum 25(OH)D levels and caries experience in U.S. children [44].

In the case of DDEs (developmental defects of enamel), studies on the role of vitamin D in the development of enamel defects are limited. However, there have been several suggestions in the literature that reduced vitamin D levels may impact the mineralization of enamel.

As the mineralization of deciduous teeth and first permanent molars begins during the prenatal period, several studies have focused on maternal vitamin D levels during the second and third trimesters of pregnancy. Among the nine included articles, five evaluated vitamin D status in pregnant women. However, only one study demonstrated a significant association between maternal vitamin D levels and enamel defects in offspring. In a longitudinal study, Børsting et al. followed 176 mother–child pairs and assessed maternal serum 25-hydroxyvitamin D levels during pregnancy, classifying them as insufficient (<50 nmol/L) or sufficient (≥50 nmol/L). Their findings revealed that lower maternal vitamin D levels at gestational weeks 18–22 were significantly associated with a greater number of teeth affected by MIH in children aged 7–9 years (adjusted RR = 1.82) [22]. A similar study conducted by van der Tas et al. found no significant relationship between maternal serum 25(OH)D levels measured at mid-gestation or in cord blood and the likelihood of 6-year-old children having at least one tooth affected by MIH or HSPM [11]. Mortensen et al. assessed s-25(OH)D levels in early and late pregnancy as well as at birth. In a cohort with relatively high s-25(OH)D concentrations and generally healthy mothers and children, the median levels were recorded at 65.0 nmol/L in early pregnancy, 79.2 nmol/L in late pregnancy, and 45.1 nmol/L in cord blood. Nevertheless, no association was found between vitamin D status during pregnancy or in cord blood and the occurrence of HSPM [20]. Kunisch et al. conducted the only study indicating that elevated serum 25(OH)D concentrations were significantly linked to a lower prevalence of MIH in 10-year-olds and a reduced number of hypomineralized teeth. Moreover, children with higher serum 25(OH)D levels exhibited significantly fewer restorations related to dental caries [27]. Another research performed by Arponen et al. aimed to compare the effects of daily vitamin D supplementation (10 μg vs. 30 μg) during the first two years of life on oral health at age 6–7 years. Conducted as a randomized, double-blind clinical trial with 975 healthy infants, the follow-up study included 123 children who underwent oral examinations. The study concluded that 10 μg of daily vitamin D3 supplementation is sufficient for maintaining good oral health in healthy children under 2 years in the Northern Hemisphere but there is no association between vitamin D supplementation and the development of enamel defects [25].

Some studies show a connection between vitamin D and enamel development (DDEs), while others do not, due to various methodological differences. Cross-sectional studies, such as Børsting et al. [24], can identify correlations but fail to establish causality, making it difficult to confirm whether low vitamin D levels directly cause molar incisor hypomineralization (MIH). Longitudinal studies, like Børsting et al. [25], help track vitamin D’s impact over time, but they may miss critical stages of enamel formation. Variations in how vitamin D levels are measured contribute to inconsistencies, as serum 25(OH)D levels fluctuate due to factors like sun exposure, diet, and supplementation. For example, Kühnisch et al. [30] found a negative correlation between high vitamin D levels and MIH, but seasonal differences could have influenced the results. The source of vitamin D—whether from sunlight, diet, or supplements—also affects study outcomes, as shown by Reed et al. [31], which focused on prenatal vitamin D and enamel hypoplasia. Population differences play a role in this, such as age and geographic location; studies in northern regions may observe more pronounced effects due to limited sunlight exposure, as seen in the Tromsø study. Confounding factors like calcium, magnesium, and phosphorus levels may obscure the vitamin D–enamel relationship, as these minerals are crucial for enamel mineralization, as demonstrated by Arponen et al. [28]. The dosage and duration of vitamin D exposure vary across studies, with long-term supplementation showing stronger effects on enamel, as seen in the 2-year intervention in Arponen et al. [28]. Finally, genetic factors and seasonal fluctuations in vitamin D levels further complicate results, as seen in studies by Reed et al. [31] and Kühnisch et al. [30], emphasizing the need for standardized and controlled approaches.

Several limitations must be considered in this review. The studies often had small sample sizes, which may affect the generalizability and statistical power of their findings. The lack of standardized methods for assessing vitamin D status and dental health outcomes introduces variability and limits comparability across studies. Additionally, important data, such as the dosage and duration of vitamin D supplementation, were not consistently reported, which could influence results. Most studies were observational, making it difficult to establish a causal relationship between vitamin D levels and enamel defects. Given the mixed findings and the influence of confounding factors—such as nutrition, health status, and sunlight exposure—future research should adopt more robust longitudinal designs, control for relevant confounders, and assess vitamin D status during critical developmental stages. Moreover, genetic factors influencing enamel formation, such as gene polymorphisms related to vitamin D metabolism, absorption, and receptor activity, warrant further investigation, though these mechanisms remain a topic for future studies. Another limitation of this review is that the search was restricted to studies published within the past 10 years.

## 5. Conclusions

Considering any limitations of the present study, based on the analyzed material, it can be concluded that there is insufficient evidence to establish a clear link between vitamin D levels during the prenatal period and after birth and the occurrence of developmental enamel defects. However, due to the limited number of studies conducted on this topic, further research is necessary.

## Figures and Tables

**Table 1:** Main characteristics of the included studies.

Author/Year/County	Study Design	Sample Size	Age of Participants	Vit. D Status Evaluation	Type of Assessed DDE
Kühnisch et al./2015/Germany [30]	Cohort (birth cohort)	1048	Follow-up visits were scheduled at 6 months, 1 year, and 18 months, and at 2, 4, 6, and 10 years of age.	Serum 25(OH)D concentrations were measured at 10 years of age.	MIH
Reed et al./2017/USA [31]	Cohort (birth cohort)	37 mother-child pairs	Mean age of children was 3.6 ± 0.9 years old.	Each pregnant woman had eight monthly 25(OH)D concentration measurements taken from 12 to 40 weeks of gestation.	Enamel hypoplasia
van der Tas et al./2018/Netherlands [32]	Cohort (birth cohort)	4750; 3406; 3983	Pregnant mothers; newborns; 6-year-old children.	Measurements were taken at three time points: mid-gestation in maternal blood, at birth in umbilical cord blood, and at 6 years of age in children’s blood.	HSPM, MIH
Dekkerhus/2020/Norway [26]	Cross-sectional study	708	16–17 years old.	One measure point during the investigation at age 16–17 y.o.	MIH
Schroth et al./2021/Canada [21]	Cohort (birth cohort)	207 mother–child pairs	Pregnant mothers; 12-month-old children	One measure point during second or early third trimester.	Enamel hypoplasia
Beckett et al./2022/New Zealand [29]	Cohort (birth cohort)	100	0–5 months old (vit. D); 6.6 years (SD ± 0.6) (dental assessment).	Evaluated three times (third trimester of pregnancy; at birth; at 5 months old).	Demarcated opacities, diffuse opacities
Børsting et al./2022/Norway [25]	Cross-sectional	176 mother-child pairs	Pregnant mothers and their children aged 7–9 years old.	Two measure points during pregnancy (gestational weeks 18–22 and 32–36).	MIH, HSPM
Mortensen et al./2022/Denmark [27]	Cohort study (birth cohort)	1241	Pregnant mothers and their 4-year-old children.	Three measurements were taken: during early pregnancy (<20 weeks), late pregnancy (≥20 weeks), and in umbilical cord blood.	HSPM
Arponen et al./2023/Finland [28]	Cohort	123	6–7 years old.	One measure point during the investigation at age 6–7 y.o.	MIH
Børsting et al./2024/Norway [24]	Cross-sectional study	101	7–9 years old.	One measure point during the investigation at age 7–9 y.o.	MIH

MIH—molar–incisor hypomineralization; HSPM—hypomineralized second primary molar.

**Table 2:** Main findings of the included studies.

Author	Outcome	DDE Relation to Vitamin D Status	Significance Level	Conclusions
Kühnisch et al., 2015 [30]	MIH	Individuals with higher 25(OH)D levels exhibited fewer hypomineralized teeth, and each 10 nmol/L increase in serum 25(OH)D concentration was associated with a significantly reduced odds ratio of developing MIH.	(OR = 0.96 per 10 nmol/L; *p* = 0.015). (OR = 0.89; *p* = 0.006)	Lower serum vitamin D levels were linked to an increased likelihood of developing MIH.
Dekkerhus, 2020 [26]	MIH	Participants with severe MIH exhibited lower 25-hydroxyvitamin D levels.	The results were not statistically significant.	Vitamin D levels are not connected with the prevalence of MIH.
Arponen et al., 2023 [28]	MIH	Developmental dental defects (DDEs) were observed in 39% of participants receiving the 10 μg/day vitamin D3 intervention and in 53% of those in the 30 μg/day group. MIH was present in the dentition of 13% of children in the 10 μg/day intervention group and 14% of participants in the 30 μg/day group.	DDE: (χ^2^(1) = 2.639, *p* = 0.104) MIH: (χ^2^(1) = 0.06, *p* = 0.807)	No associations were found between vitamin D intervention group in infancy and oral health or the presence of DDE.
Børsting et al., 2024 [24]	MIH	A greater proportion of children were affected by MIH in the insufficient vitamin D group compared to the sufficient group (+11.7% vs. +8.4%). Additionally, children in the insufficient group had a higher average number of MIH-affected teeth (+0.4).	MIH showed no statistically significant associations with having insufficient or lower vitamin D levels.	Vitamin D status was not significantly associated with the prevalence and number of teeth affected by MIH among 7–9-year-old children in Norway.
Mortensen et al., 2022 [27]	HSPM	No correlation was identified between continuous cord serum 25(OH)D levels and the occurrence of HSPM.	0.998 (95% CI 0.992–1.004, *p* = 0.501)	No link was found between vitamin D status during pregnancy or in cord blood and the occurrence of HSPM.
van der Tas et al., 2018 [32]	MIH, HSPM	No association was found between fetal 25(OH)D concentrations and the presence of HSPMs or MIH. In 6 year olds, a higher 25(OH)D concentration in umbilical cord blood resulted in neither lower odds of having HSPM or MIH. No higher 25(OH)D concentrations at the age of six to be associated with a significant change in the odds of having HSPM or MIH	(OR 1.02 per 10 nmol/L higher 25(OH)D, 95% CI: 0.98–1.07) (OR 1.05 per 10 nmol/L increase, 95% CI: 0.98–1.12) (OR 1.05, 95% CI: 0.98–1.13) (OR 0.95, 95% CI: 0.84– 1.07) (OR 0.97, 95% CI: 0.92–1.02) (OR 1.07, 95% CI: 0.98–1.16)	No associations with the presence of HPSMs or with MIH at the age of six.
Børsting et al., 2022 [25]	MIH, HSPM	Among children with MIH, a higher number of affected teeth were observed in those whose mothers had insufficient vitamin D levels between gestational weeks 18–22 compared to those with sufficient maternal vitamin D. However, no such differences were noted in children affected by HSPM.	(*p* = 0.01) (*p* = 0.32)	Insufficient maternal serum vitamin D levels during mid-pregnancy were linked to a greater number of affected teeth in children with MIH at ages 7–9.
Reed et al., 2017 [31]	DDE	Maternal 25(OH)D (12–40 weeks of gestation) with enamel hypoplasia = 32.1 ± 13.6 ng/mL without enamel hypoplasia = 33.6 ± 12.8 ng/mL	*p* = 0.76	This was a preliminary study which suggests a need for more investigation.
Schroth et al., 2021 [21]	DDE	Although mothers of infants with enamel hypoplasia had lower average 25(OH)D concentrations, the differences were not statistically significant compared to those of mothers whose children did not present with enamel hypoplasia.	*p* = 0.072	No significant association between 25(OH)D concentration and enamel hypoplasia.
Beckett et al., 2022 [29]	DDE	DDEs were detected in 5%, 30% and 32% of patients with deficient Vit. D level (<30 nmol) in maternal blood, cord blood and infant blood, respectively. DDEs were detected in 58%, 36% and 58% of patients with sufficient Vit. D level (>50 nmol) in maternal blood, cord blood and infant blood, respectively.	IRR 0.37–0.69, *p* > 0.05	No associations were observed between 25(OH)D levels at any time point and the development of any type of dental enamel defect.

**Table 3:** Risk of bias assessment of included studies using Newcastle–Ottawa Scale.

Author, Year	Selection	Comparability	Outcome	Total
Kühnisch et al., 2015 [30]	****	*	**	7
Reed et al., 2017 [31]	***	**	***	8
van der Tas et al., 2018 [32]	***	**	**	7
Dekkerhus, 2020 [26]	**	*	**	5
Schroth et al., 2021 [21]	***	**	***	8
Beckett et al., 2022 [29]	**	**	***	7
Børsting et al., 2022 [25]	**	**	***	7
Mortensen et al., 2022 [27]	***	**	**	7
Arponen et al., 2023 [28]	**	*	***	6
Børsting et al., 2024 [24]	**	*	**	5

## Data Availability

Not applicable.
